# Constructing a questionnaire for assessment of awareness and acceptance of diversity in healthcare institutions

**DOI:** 10.1186/1472-6963-13-145

**Published:** 2013-04-22

**Authors:** Azita Emami, Jalal Safipour

**Affiliations:** 1 Karolinska Institutet, Neurobiology, Care Science and Society, Aging Research Centre, Stockholm, Sweden; 2 Seattle University, College of Nursing, Seattle, WA, USA; 3 Faculty of Nursing, University of Alberta, Alberta, Canada

**Keywords:** Assessment, Culture, Cultural awareness, Diversity, Environment, Healthcare, Questionnaire development, Sweden

## Abstract

**Background:**

To develop a healthcare environment that is congruent with diversity among care providers and care recipients and to eliminate ethnic discrimination, it’s important to map out and assess caregivers’ awareness and acceptance of diversity. Because of a lack of standardized questionnaires in the Swedish context, this study designed and standardized a questionnaire: the Assessment of Awareness and Acceptance of Diversity in Healthcare Institutions (AAAD, for short).

**Method:**

The questionnaire was developed in four phases: a comprehensive literature review, face and content validity, construct validity by factor analysis, and a reliability test by internal consistency and stability assessments.

**Results:**

Results of different validity and reliability analyses suggest high face, content, and construct validity as well as good reliability in internal consistency (Cronbach’s alpha: 0.68 to 0.8) and stability (test-retest: Spearman rank correlation coefficient: 0.60 to 0.76). The result of the factor analysis identified six dimensions in the questionnaire: 1) Attitude toward discrimination, 2) Interaction between staff, 3) Stereotypic attitude toward working with a person with a Swedish background, 4) Attitude toward working with a patient with a different background, 5) Attitude toward communication with persons with different backgrounds, 6) Attitude toward interaction between patients and staff.

**Conclusion:**

This study introduces a newly developed questionnaire with good reliability and validity values that can assess healthcare workers’ awareness and acceptance of diversity in the healthcare environment and healthcare delivery.

## Background

Globalization and immigration are changing not only the demographic composition of society, but also the pattern of social relationships and social interaction among individuals. In a multicultural society, members are challenged to preserve their own identity when interacting with other members with different socio-cultural backgrounds [1]. Sweden, as a part of the European Union, is currently undergoing this transition toward multiculturalism and diversity because of rapid and expansive immigration into the country.

One of the main areas of inquiry of public health research is to investigate the health and well-being of the individual members of society in order to provide an environment that is inclusive, supportive, and free from discrimination for all members [2]. Sufficient knowledge about structural and individual mechanisms that impact user-friendly and culturally sensitive care is lacking, which may result in discrimination in Swedish healthcare institutions [3-5]. Cultural competence is a skill set that can help healthcare providers create culturally sensitive and user-friendly care services for people with diverse backgrounds [6].

Evidence shows that most misunderstandings between healthcare providers and patients with different cultural backgrounds arise from providers’ lack of understanding, cultural awareness, cultural knowledge, and flexibility [7]. In addition, providers often have a one-dimensional biomedical perspective rather than a holistic perspective in caring for patients [8-10]. One way of assessing the awareness and acceptance of diversity and how it impacts the interactions and communications taking place in healthcare settings is to use a questionnaire to study the self-reported perceptions and experiences of individuals who interact in healthcare institutions [11].

A valid and reliable questionnaire for investigating awareness and acceptance of diversity among staff working in healthcare settings has been lacking in Sweden. Another research project attempted to provide a valid and reliable cultural competence questionnaire but the result indicated weak validity and reliability, and a lack of consistency in the structure of the scale [10]. Therefore, this study aimed to construct a more valid and reliable questionnaire to map out and assess awareness and acceptance of diversity among healthcare staff, based on a study at two elderly care institutions in Stockholm.

This project was a part of a larger research investigation exploring how healthcare institutions in Sweden address diversity and equal rights in regard to the workplace environment and healthcare delivery. The main objective for the larger project was to enhance healthcare institutions’ awareness of the importance of a perspective that is sensitive to culture and diversity, and to prevent ethnic discrimination in the work environment as well as in the delivery of care. The framework for constructing the questionnaire was based on the notion that the society and the individual create each other; culture is learned and shared and is a result of intersections of different qualities, such as gender, socio-economic status, education, as well as historical, geographical, political, and societal circumstances [12,13]. In this way, individuals actively create their collective experience through social interactions in social contexts.

Many nursing science researchers have used the concept of cultural competence in studies that focus on nurses´ care of patients with different cultural backgrounds [8,14,15]. The concept is complex and many definitions are given in the literature. Moreover, by not addressing the complexity of cultural competence and what it should stand for, some of these studies may unintentionally reinforce stereotypes by reiterating certain cultural norms and distinctions as universal predictors for people’s behavior [16]. In constructing the questionnaire for this project, we attempted to go beyond the concept of cultural competence and to shed light on awareness and acceptance of diversity and its impact on the social interactions between individuals with diverse backgrounds who work in healthcare institutions.

The questionnaire was aimed at the entire healthcare institution, i.e., including both care-providing and non-care-providing staff and their relationships with patients and the patients’ significant others. This questionnaire was intended to serve as a tool for obtaining data concerning awareness and acceptance of healthcare providers as they interact with people with diverse backgrounds.

## Methods

### Construction of the questionnaire

**Figure 1:**
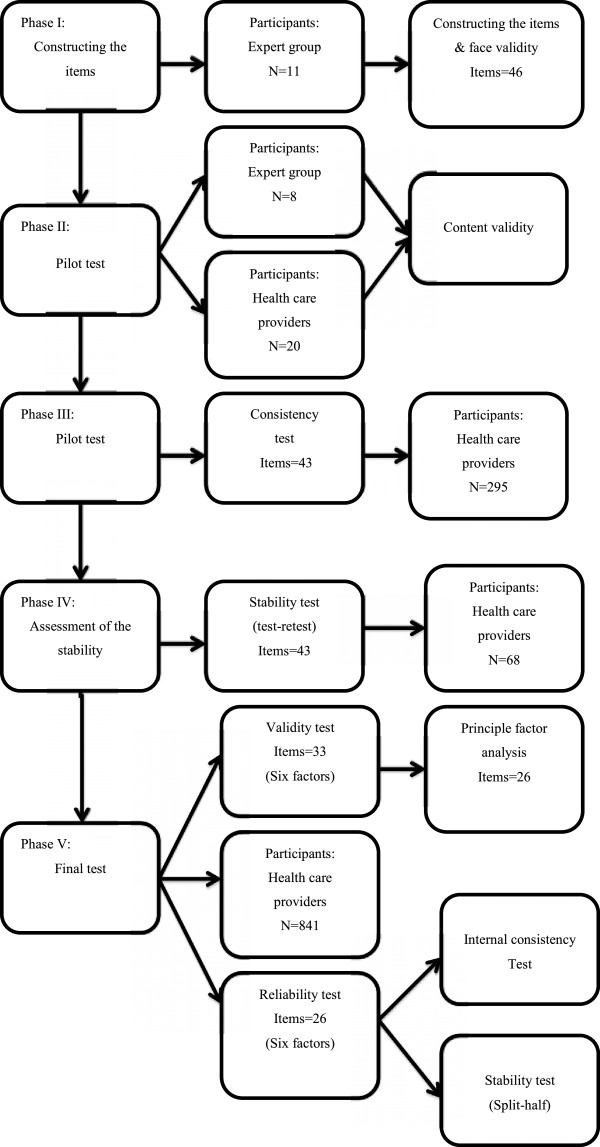
Study process, from item selection to testing reliability and validity of the scale.

The Assessment of Awareness and Acceptance of Diversity in Healthcare Institutions questionnaire (AAAD) was developed in several phases to ensure validity and reliability. The first phase was selection of items by experts. The reliability and validity of the questionnaire was examined in several pilot tests with different scale construction methods, including face, content, and construct validity. These were conducted by using expert groups, factor analysis, and a reliability test on internal consistency and stability (Figure 1).

This research received ethical approval from the Research Ethics Committee at the Karolinska Institutet-Stockholm (reference 2009/463-31). Permission to distribute the questionnaire was obtained from the Nursing Homes and Home-based Care settings. A cover page was added to the questionnaire describing the project and stressing that the participation was voluntary. Questionnaires were completed anonymously and the questions were constructed to preserve confidentiality (personal details such as names, personal numbers, and addresses were not included). A code known only to the research team was included on each questionnaire in order to identify the workplace not individuals. In other words, when the questionnaires were returned in the envelopes, not even the research team was able to identify the participants.

### Statistical analysis

Several statistical analyses were applied in the last stages of the pilot tests of constructed items for testing reliability and validity of the questionnaire. The items were constructed in a Likert-type scale and the items could be scored individually as well as summed in groups. The items phrased negatively were reverse coded. Reliability of the questionnaire was tested using two methods: an internal consistency test (with a Cronbach’s alpha coefficient) and a stability test (either test-retest or split-half test with a Spearman-Brown coefficient or a Guttman Split-Half coefficient).

Test-retest reliability refers to whether a questionnaire yields a consistent measurement over time [17]. To estimate the test-retest reliability of the AAAD subscales and items, a Spearman correlation was used for all subscales and single items, and a percentage of agreement (PA) was used for the subscales and single items with a low Spearman correlation. The closer the correlation coefficient (r) is to 1.0, the stronger the correlation [17]. A PA of 80 is usually regarded as a good level of test-retest reliability.

Internal consistency describes estimates of reliability based on the average correlation among items within a test or subscales [17-19]. Chronbach’s alpha coefficients were used to estimate the internal consistency of the subscales in the questionnaire.

Factor analysis was applied to assess the validity of the questionnaire and to identify items underlying each factor [20]. The Kaiser-Meyer-Olkin (KMO) and Bartlett’s Test was applied to test factorability of the data. A KMO value closer to 1 is good and 0.6 is acceptable. Items with KMO values less than 0.5 or low communality are usually considered as items that can be dropped from the analysis.

## Results

### Phase I

#### Initiating the construction of the questionnaire—face validity

First, a literature review on subjects related to the research area was carried out to provide an initial basis for selecting items (in Swedish) to be included in the questionnaire. An expert group was established, consisting of four researchers in transcultural care, one anthropologist, one sociologist, one social welfare officer, one deputy assistant undersecretary, the parliamentary commissioner for discrimination, one expert from the Swedish Integration Board (officially called the “Discrimination Ombudsman” (DO)), and one person from the Swedish Center Against Racism (CMR). The first draft of a questionnaire including 73 items was given to the expert group, and then revised accordingly, with the expert group supplying new comments by email. For example, the expert group raised possible negative associations that the word discrimination might arouse and suggested replacing it with equal rights.

Generally, comments from this group suggested that subjects such as discrimination, cultural awareness, and ethnicity needed to be dealt with delicately and sensitively. The group surmised that people would be usually anxious about and reluctant to talk openly and to answer questions about these issues. Another problem during the process was the use of verbiage and phrases that could be comprehended by people in all the different professional categories with different occupational, educational, social, economic, and ethnic backgrounds as well as by people with different native languages that we planned to include in the study population. The experts emphasized that the terminology used in the questionnaire had to be straightforward and immediately understandable by all respondents and should be as short as possible to prevent respondent exhaustion. The items were revised based on these comments and suggestions for using the most appropriate phrasings for constructing the items of the questionnaire and the written information and guidelines for filling out the questionnaire.

The questionnaire had three basic parts. The first part contained socio-demographic questions, which did not need to go through the process of assessing validity and reliability. The second part of the questionnaire was directed towards all categories of staff who worked in a healthcare institution, while the third part was only for care providers. The second and third parts had 46 items and concerned communication, attitudes, and discrimination in relation to the patients and their significant others in care-providing situations. Thus only these 46 items were processed for future validity and reliability assessment.

### Phase II

#### Pilot studies—content validity

In the first pilot study, the questionnaire was sent to healthcare scientists in a related field. They were given an overview of the theoretical framework of the study and asked to answer the questions as well as to comment on the items in particular and the questionnaire in general. Participation was voluntary. Two recipients declined participation; one of them stated that the questions were too sensitive to discuss in public arena, and the other did not have time. At least 8 scientists evaluated the items, showing that the terminology used in many of the items, especially the word discrimination, was indeed too loaded. Participants emphasized that they became annoyed while reading the questions. Some expressed fear over sharing their knowledge about existing problems related to discrimination in their workplace, and many expressed that the items related to discrimination in the workplace were confusing. They suggested that some of the items were difficult to understand, and most participants said that it was too time-consuming to answer the questionnaire.

Based on the comments received in this phase, a “think-aloud” process was performed in the next step of revising the questionnaire. A researcher experienced in constructing similar questionnaires was asked to read the instructions and items aloud. She then shared her thoughts as she read and answered items to the research team. The use of this method resulted in many constructive comments concerning wording, understanding, and layout.

As a result, the questionnaire was shortened and the wording was simplified to ensure that respondents could easily understand the items and complete the questionnaire quickly. For example, the words *ethnicity* and *discrimination* were deleted from the questionnaire since they seemed to contribute to anxiety and confusion. The word *ethnicity* was replaced by the phrase “people with a Swedish background and people with backgrounds in countries other than Sweden.”

In this phase, based on the expert views, the items were categorized as follows:

a) social interaction between staff and between staff and patients, b) attitude toward communication with people with different backgrounds, c) constitution of stereotypes, and d) discrimination in the workplace.

The revised questionnaire was sent to 20 people who were working in a healthcare setting to identify whether or not the items could be understood by the target population. Based on the comments gathered from the second piloting, the questionnaire was again revised. This time three items about stereotypes in general were deleted, and the total number of questions was decreased to 43.

### Phase III

#### Consistency of items

The revised questionnaire was distributed to all of the staff working (295 individuals) in healthcare setting #1 for the third piloting round. The 295 copies of the questionnaire, which included an information letter about the study, instructions for completing the questions, information flyers for all the staff, and special delivery boxes for the questionnaire were delivered to the heads of each unit in the participating healthcare setting. In total, 205 (69%) of the 295 recipients completed and returned the questionnaires. None of the head nurses heard any negative comments from the staff about wording, the concepts, or items.

The results of the pilot study showed a good internal consistency with a Cronbach’s alpha range of 0.62 to 0.85 for most the subscales after deleting items with low correlation with other item groups.

### Phase IV

#### Assessment of the stability of the items

Based on the results of the internal consistency test, some minor revisions also were made to the questionnaire including rewording of an item and the addition of one heading to add more clarity. Furthermore, a test-retest was conducted to assess the reliability of the questionnaire. Prior to data collection for the test-retest, the researchers held an information seminar for the head nurses at the selected elderly care unit and another for all the staff at the selected unit in healthcare setting #2, which would be included in the main study. Information flyers were also delivered to the unit to increase the awareness of the potential respondents about the study. The questionnaire, including the information letter with background information and instructions for completing the questionnaire, were distributed to the total staff (119 people) who were working during one specific shift at the selected elderly care unit.

All of the respondents received two copies due to the fact that researchers could not identify the sample population after delivering the questionnaires for follow-up. The questionnaire was handed out together with the instruction to answer the questionnaire twice with a maximum seven-day interval between the response days. A total of 98 individuals (88%) filled out the questionnaire at least once while 68 people (61%) filled it out twice.

The Cronbach’s alpha coefficients in this study ranged, for subscales, from 0.65 to 0.81. The items with the Spearman correlation coefficient for test-retest that ranged from 0.6 to 0.91 were retained in the questionnaire. In this phase, two items with low correlation coefficients (0.39 and 0.50) were excluded from the questionnaire. The expert group gathered one more time and the items were reduced to 33 based on statistical results from phases III and IV.

### Phase V

#### The final validity and reliability test

In the last phase of constructing this questionnaire, all the remaining items were tested for validity and reliability. A total of 841 people out of 1016 (83%) who worked in healthcare setting #2 in Stockholm participated in this study; 85% of those were care-providing staff (nurses, physiotherapists, doctors, and psychologists). Mangers distributed the questionnaires and respondents deposited them in a box provided for this purpose. The unit selected for the test-retest study was excluded from participating in the main study. The percentage of females was 83.9% and 47.5% of them had been born in a country other than Sweden (see Table 1).

**Table 1:** Demographic characteristics of the sample population

**Variable, n (%)**	n=841*
Gender (female) n=821*	689 (83.9)
Age: n= 819*	
>−25	88 (10.7)
25-35	172 (21.0)
35-45	215 (26.3)
45-55	205 (25.0)
55-<	139 (17.0)
Born not in Sweden, n=819*	389 (47.5)
Profession: n=798*	
Health care staff	678 (85.0)
Administrative staff	49 (6.1)
Food staff	15 (1.9)
Others:	56 (7.0)

*Due to missing data the sum is not 841.

A validity test was done by means of principle factor analysis for all items remaining in the questionnaire. After communality check and KMO analysis (cut-off 0.6), some items were dropped from the questionnaire and 26 items remained for final factor analysis. The result of the factor analysis for evaluating dimensionality of the items indicated six factors among the 26 items (Table 2). The KMO was 0.82 for all items and the Bartlett’s Test of Sphericity was significant (Chi-square=3675,46; Df=325, p<0.001).

**Table 2:** The rotated component matrix for 26 items

**Factors/Items**	**1**	**2**	**3**	**4**	**5**	**6**
1	**,764**	-,028	-,181	,043	-,015	-,109
2	**,750**	-,047	,056	-,031	-,019	-,072
3	**,745**	,111	-,088	-,031	-,005	,046
4	**,735**	-,032	-,056	,013	-,114	-,068
5	**,583**	-,073	-,169	-,075	,114	,244
6	**,551**	,013	-,418	-,154	-,124	-,134
7	**,452**	-,103	-,471	,030	-,151	-,151
8	-,024	**,702**	-,028	,124	-,099	-,067
9	,062	**,688**	-,121	,163	,229	,189
10	-,127	**,671**	-,085	,003	-,241	-,120
11	-,063	**,631**	,092	,123	,139	,056
12	,026	**,629**	-,084	,231	,260	,307
13	,081	**,569**	,287	,089	,048	,161
14	-,014	**,507**	,366	,173	,022	,048
15	-,127	,004	**,781**	,089	,064	-,015
16	-,034	,027	**,759**	-,033	,124	,056
17	-,300	,049	**,704**	,096	,009	,096
18	,089	,204	,114	**,747**	,179	,035
19	,005	,249	,056	**,738**	,104	,166
20	,000	,143	,072	**,737**	,196	,113
21	-,197	,061	-,023	**,630**	-,127	,009
22	-,093	-,027	,072	,089	**,808**	-,023
23	-,035	-,008	,110	,150	**,793**	,102
24	-,034	,422	,133	,030	**,611**	-,038
25	-,019	,115	,074	,099	-,038	**,804**
26	-,111	,085	,134	,149	,073	**,773**

#### Factor one: attitude toward discrimination

The first factor contained items regarding *attitude toward discrimination* in the workplace. This factor contained seven items. The inter-item correlation coefficient revealed that all correlations were significant at a level of <0.001 (Table 3). The result indicated that all seven items underlie one component (factor), the correlations between items are positive and significant, and the total percentage of variance explained by this factor is 26.80%.

**Table 3:** Correlation matrix of seven items for factor about discrimination in the workplace

Items*	**1**	**2**	**3**	**4**	**5**	**6**	**7**
I think that staff are treated with the same respect at my workplace irrespective of the country they come from	**1,00**						
I think that tasks are distributed equally among staff at my workplace irrespective of whether they come from different countries.	,46	**1,00**					
I think that workmates with a background other than Swedish get the same degree of information about important issues as workmates with a Swedish background.	,40	,39	**1,00**				
I think that staff at my workplace have the same influence independent of their background.	,48	,63	,46	**1,00**			
I think that staff with backgrounds from countries other than Sweden receive breaks during lunch as often as staff with a Swedish background.	,37	,25	,400	,26	**1,00**		
I think that staff with backgrounds from countries other than Sweden are more often blamed if things go wrong in work than staff from Sweden.	,43	,35	,38	,37	,28	**1,00**	
I think that workmates with a background other than Swedish are more controlled than workmates with a Swedish background.	,40	,26	,25	,30	,32	,56	**1,00**

All correlations are significant at *p*<.001.

*Some items reverse-coded based on consistency of the measurement.

A reliability test was conducted with respect to internal consistency, which was tested by Cronbach’s alpha, and stability (split-half method, which is equal to test-retest), which was tested by Spearman-Brown coefficient and Guttman split-half coefficient. The Cronbach’s alpha was 0.81, the Spearman-Brown coefficient was 0.72, and the Guttman split-half coefficient was 0.73, which all suggest high stability and internal consistency of the seven items in factor one.

#### Factor two: interaction between staff

The second factor identified in factor analysis also contained seven items. This factor related to attitude toward working with the people with different ethnic/cultural backgrounds. All the items in factor two positively correlated and all the correlations were significant, suggesting they were in fact measuring one component in the questionnaire (Table 4), with 16.43% of total variance for these seven items.

**Table 4:** Correlation matrix of seven items for factor about Interaction between staff

Items*	**1**	**2**	**3**	**4**	**5**	**6**	**7**
I find it interesting to work with people with backgrounds from different countries.	**1,00**						
It is more difficult to communicate with staff whose background is from a country other than my own.	,34	**1,00**					
I learn new things about life from cooperating with people from other countries.	,46	,29	**1,00**				
If I can choose, I prefer to cooperate with a staff member whose background is from the same country as me rather than with staff from other countries.	,33	,50	,28	**1,00**			
Misunderstandings occur more often when I collaborate with colleagues whose backgrounds are from countries other than my own.	,33	,67	,24	,44	**1,00**		
A person with a background other than Swedish is uninterested in learning about Swedish values, norms, and habits.	,32	,33	,30	,32	,34	**1,00**	
People with a background other than Swedish don’t take as much responsibility in their work as a person with a Swedish background.	,26	,30	,27	,31	,31	,530	**1,00**

All correlations are significant at *p*<.001.

*Some items reverse-coded based on consistency of the measurement.

The internal consistency of items, which was measured by Cronbach’s alpha for all seven items, was 0.80. The Spearman-Brown coefficient was 0.78 and the Guttman split-half coefficient was 0.77, suggesting reliability of the items.

#### Factor three: stereotypic attitude toward working with people with a Swedish background

Factor analysis indicated this factor contained three items, which dealt with stereotypic attitudes toward working with people with a Swedish background. The correlations between the items were positively significant and 11.31% of variance was explained by these three items. The correlation matrix was shown in Table 5.

**Table 5:** Correlation matrix of three items for factor about stereotypic attitudes

Items*	**1**	**2**	**3**
People with a Swedish background are more often individualists than people with backgrounds from other countries.	**1,00**		
In general, people with Swedish background in Sweden are uninterested in learning about people’s values in different countries.	,53	**1,00**	
People with a Swedish background have an attitude of superiority towards people from other countries.	,54	,43	**1,00**

All correlations are significant at *p*<.001.

*Some items reverse-coded based on consistency of the measurement.

A reliability test was also performed to examine the stability and internal consistency of the items. The Cronbach’s alpha was 0.70, which confirmed the consistency of the items. The Spearman-Brown coefficient was 0.74 and the Gutman split-half coefficient was 0.66.

#### Factor four: attitude toward working with patients with a different background

This factor contains four items, which dealt with attitudes toward providing care for patients with different backgrounds. All correlations between items are positive and significant (see Table 6). The total variance explained by this factor was 14.60%.

**Table 6:** Correlation matrix of four items for factor about attitude toward difficulties working with patients with a different background

Items*	**1**	**2**	**3**	**4**
It is more difficult to meet the needs of patients whose background is from a country other than my own.	**1,00**			
Misunderstandings occur more often when I care for patients whose background is from a country other than my own.	,58	**1,00**		
It is more difficult to understand a patient’s preferences for care if she/he comes from a country other than my own.	,50	,53	**1,00**	
It is as easy to understand and communicate with patients from another country as it is with patients from my own country.	,30	,28	,34	**1,00**

All correlations are significant at *p*<.001.

*Some items reverse-coded based on consistency of the measurement.

The Cronbach’s alpha, indicating consistency of the items for all four items, was 0.75. The Spearman-Brown coefficient was 0.71 and the Guttman split-half coefficient was 0.71. Both indicators suggested high stability of the identified factor.

#### Factor five: attitude toward communication with people with different backgrounds

A total of three items were contained in this factor with factor loading range from 0.6 to 0.8. The percentage of variance explained by this factor was 8.77%. The items appeared significantly correlated together and, as the correlation matrix shows (see Table 7), the correlations are positive.

**Table 7:** Correlation matrix of three items for factor about communication with people with a different background

Items*	**1**	**2**	**3**
I think education about communication between people with backgrounds from different countries at my workplace would be a good thing.	1,00		
There is a need for occasions to discuss issues about people with background from different countries at my workplace.	,49	1,00	
There are no communication difficulties at all at my workplace because the staff have backgrounds from different countries.	,34	,40	**1,00**

All correlations are significant at *p*<.001.

*Some items reverse-coded based on consistency of the measurement.

The indicator for consistency of the items was acceptable and fair with value of 0.68 (Cronbach’s alpha). The stability of this subscale was also acceptable with a Spearman-Brown coefficient of 0.61 and Guttman split-half coefficient of 0.54.

#### Factor six: attitude toward interaction between patient and staff

This factor assessed whether patients treat staff equally regardless of their background. Two items remained in this factor and the correlation between them was positive and significant at 0.44. The variance was explained by this factor was 5.15%. The items included in this factor were:

1. In my workplace, it sometimes occurs that patients treat care providers from other ethnic backgrounds than their own worse than the way they treat care providers with the same ethnic background.

2. In my workplace, it sometimes occurs that patients’ family members treat care providers from other ethnic backgrounds than their own worse than the way they treat care providers with the same ethnic background.

Although there were only two items in this factor, the Cronbach’s alpha, which is usually low for factors with few items, was acceptable with value of 0.61. The Spearman-Brown coefficient and Guttman split-half coefficients were also fair and acceptable for stability of the items with a value of 0.61.

#### The final questionnaire

The final questionnaire was divided into seven sections for use among health care providers. Two parts were only for care-providing staff (nurses, physiotherapists, doctors, and psychologists). The first section of the instrument asked about general socio-demographic information. The second section included 26 items in six factors. The first factor included seven items dealing with discrimination in the workplace. The second factor containing seven items was about the interaction of staff in the workplace. The third factor, dealing with stereotypic attitudes, contained three items. The fourth factor, with four items, was about attitudes toward working with patients with different backgrounds. The fifth factor with three items was about communication difficulties with people with different backgrounds. And the final factor concerned attitudes toward the interaction of patients with different backgrounds and healthcare staff.

## Discussion

This paper presents the process of constructing a questionnaire to map out and assess the awareness and acceptance of diversity among staff working in healthcare institutions in Sweden. The intention of constructing the questionnaire was to illuminate the obstacles that may prevent a diverse healthcare institution from being sensitive to and congruent with diversity. The main strength of this study was to use various validity and reliability measures in constructing the questionnaire.

There is a shortage of standardized tools with high validity and reliability for assessing the level of awareness and acceptance of diversity in healthcare institutions [10]. One reason for this may be that the construction of reliable items and subscales to explore attitudes is more complex and difficult when it comes to sensitive topics such as diversity than is the case for constructing items about facts. Aspects such as ‘social desirability’ often become a problem [17,21].

The first steps in the process of constructing a questionnaire is identification of items [22-25], and the careful selection of them by conducting standardized tests, in regards to what the questionnaire is supposed to measure. One of the main concerns for assessing the individual’s perspective or attitude toward sensitive issues is that the individuals may be reluctant to express their “real” feelings. For minimizing this problem, the items needs to be selected carefully and formulated in the way the respondents are willing to answer them [22,25]. The selection of items as the most important part of the validity of the scale was the main focus of the first and second phases of this study. In this study, critical evaluation of content was made and the expert group was consulted in the process of item and sub-scale construction. As the questionnaire was revised, one of the most important concerns for the authors was to try to construct a questionnaire that was ‘user-friendly’; that is, easily understood by everyone in the target population. Furthermore, since the topics of the questionnaire were sensitive, involving moral issues, the risk of responding to the questions based on social desirability was taken into consideration. Most of the items and sub-scales refrained from investigating the behaviors or attitudes of the respondents themselves. Instead, the items posed questions that referred to the general situations and/or the respondent’s perceptions or experiences of the issues that she/he observed in her/his work.

Evidence based on response process generally comes from an analysis of individual responses and can contribute to questions about differences in meaning or interpretation of test scores among the relevant examinees. Written and spoken comments on the items in the first pilot study and the ‘think aloud study’ were analyzed and contributed to several changes in items and wording.

Several statistical analyses also were used to assess the reliability and validity of the instrument. These processes were multi-step endeavors and in each step specific analyses were performed with different sample settings to reduce the probability of bias. The results suggested that the questionnaire is valid and reliable to use in a Swedish context in the healthcare sector with dimensions regarding discrimination, interaction, stereotyping, and communication difficulties among healthcare staff and patients.

In relation to the issue of generalization of findings, the validity of the questionnaire and to what extent it can be used for utilization in different settings, it is important to consider to what degree evidence of validity based on test-criterion relations can be generalized to a new situation (for example, place, job, or education), without further study of validity in that new situation. The fact that the wording of the items is easy to understand and directed towards all categories of professions independent of their education level contributes to considering the questionnaire valid in many different settings.

It needs to be noted that a majority of the sample population in the final test were female, due to the nature of the sample population. In Sweden, almost 90% of nurses are female and the number of the nurses is higher than that of physicians [26]. We are aware that it can potentially increase the risk of bias and of validity of the questionnaire. We recommend additional testing of the questionnaire with equal numbers of men and women in order to avoid bias, if the questionnaire is to be used in other settings. We must also acknowledge that due to the lack of similar valid questionnaires/tools in Swedish, we could not examine the external validity of the questionnaire [10,27,28].

Furthermore, since the topics of the questionnaire were sensitive and involved moral issues, the risk of social desirability was taken into consideration. The problem with sensitive questions and the reluctance of individuals to respond freely to items can be minimized by extensive and clear information dissemination to the informants about the purpose of the study, their rights to withdraw their participation, and the confidentiality of responses. Most of the items and sub-scales avoided investigating the behaviour or attitudes of the respondent herself/himself. Instead the items sought responses related to the general situations, i.e., discrimination and communications in the workplace and with patients and their significant others. Furthermore this study introduced a valid questionnaire for use in the healthcare sector concerning the awareness and acceptance of diversity, discrimination, interaction, and communication among staff and patients, and is not recommended for use in different study settings with different study perspectives. Also needing to be taken into consideration is the fact that the results from attitude measurement scales do not necessarily indicate that the respondents behave or act based on how they respond [29,30]. It needs to be noted that people act differently based on different circumstances, and several other social and environmental factors may shape individuals’ behaviors [11]. Thus we recommend studies in the future use this instrument with observations to validate to what extent people act based on how they respond. By doing this the researcher could get a clearer picture of the phenomena regarding the assessment of awareness and acceptance of diversity in healthcare sectors.

## Conclusions

Improving healthcare service is always a concern of healthcare providers. Investigating and evaluating this issue is the first step in understanding the challenges arising from diversity and a lack of cultural awareness in the healthcare sector, which can impact effective healthcare services. Existing tools to measure cultural awareness of healthcare providers had proven to not be usable in a Swedish context [10]. This paper attempted to provide a valid and reliable questionnaire for use in a Swedish context. The instrument was tested with a variety of statistical tests that suggested the scale was adequate, valid, and reliable for use in a Swedish context among healthcare providers. For international utilization of this questionnaire, we recommend further contextually appropriate evaluations.

## Competing interests

We (the authors) declare that there was no conflict of interest (*financial and non-financial).* Thus our answers to the all following questions in this regard is “NO.”

Financial competing interests

• In the past five years, have you received reimbursements, fees, funding, or salary from an organization that may in any way gain or lose financially from the publication of this manuscript, either now or in the future? ***No***

• Do you hold any stocks or shares in an organization that may in any way gain or lose financially from the publication of this manuscript, either now or in the future? ***No***

• Do you hold or are you currently applying for any patents relating to the content of the manuscript? Have you received reimbursements, fees, funding, or salary from an organization that holds or has applied for patents relating to the content of the manuscript? ***No***

• Do you have any other financial competing interests? ***No***

Non-financial competing interests

Are there any non-financial competing interests (political, personal, religious, ideological, academic, intellectual, commercial, or any other) to declare in relation to this manuscript? ***No***

## Authors’ contributions

AE participated in the design of the study and critical revision of the manuscript. JS participated in drafting the manuscript and performed the statistical analyses. All authors read and approved the final manuscript.

## Pre-publication history

The pre-publication history for this paper can be accessed here:

http://www.biomedcentral.com/1472-6963/13/145/prepub
